# Structural Learning in a Visuomotor Adaptation Task Is Explicitly Accessible

**DOI:** 10.1523/ENEURO.0122-17.2017

**Published:** 2017-08-28

**Authors:** Krista M. Bond, Jordan A. Taylor

**Affiliations:** Department of Psychology, Princeton University, Princeton, NJ 08540

**Keywords:** explicit re-aiming, structural learning, visuomotor rotation task

## Abstract

Structural learning is a phenomenon characterized by faster learning in a new situation that shares features of previously experienced situations. One prominent example within the sensorimotor domain is that human participants are faster to counter a novel rotation following experience with a set of variable visuomotor rotations. This form of learning is thought to occur implicitly through the updating of an internal forward model, which predicts the sensory consequences of motor commands. However, recent work has shown that much of rotation learning occurs through an explicitly accessible process, such as movement re-aiming. We sought to determine if structural learning in a visuomotor rotation task is purely implicit (e.g., driven by an internal model) or explicitly accessible (i.e., re-aiming). We found that participants exhibited structural learning: following training with a variable set of rotations, they more quickly learned a novel rotation. This benefit was entirely conferred by the explicit re-aiming of movements. Implicit learning offered little to no contribution. Next, we investigated the specificity of this learning benefit by exposing participants to a novel perturbation drawn from a statistical structure either congruent or incongruent with their prior experience. We found that participants who experienced congruent training and test phase structure (i.e., rotations to rotation) learned more quickly than participants exposed to incongruent training and test phase structure (i.e., gains to rotation) and a control group. These results suggest that structural learning in a visuomotor rotation task is specific to previously experienced statistical structure and expressed via explicit re-aiming of movements.

## Significance Statement

Structural learning is a meta-learning phenomenon evidenced by an accelerated learning rate for novel tasks sharing the same statistics as the training task. Previous investigations suggest that this effect is driven by the implicit extraction of invariant task features. However, this interpretation contrasts with recent research showing that an explicitly accessible process, such as movement re-aiming, accounts for most of rotation learning. We investigated (1) whether structural learning in a visuomotor rotation task was explicitly accessible and (2) whether structural learning was specific to the trained perturbation structure or expressed via a general aiming heuristic. Our results suggest that structural learning in a visuomotor rotation task is specific to previously experienced statistical structure and expressed via movement re-aiming.

## Introduction

Ebbinghaus coined the term “savings” to characterize the phenomenon of faster relearning of material despite its apparent forgetting (Ebbinghaus, 1885). Structure learning is a related but distinct phenomenon; whereas savings operates over time (Ebbinghaus, 1885) and within the same input-output mapping (Harlow, 1949; Ashby, 1960), structural learning operates over parameter space and within a class of mappings (Braun et al., 2010). Instead of increasing learning rate through consolidation, structural learning abstracts relationships through experience within the parameter space of a task, which reduces the dimensionality of the hypothesis space.

**Figure 1.**
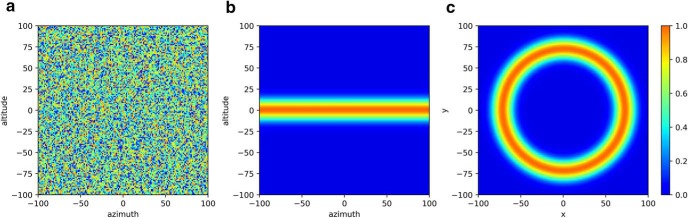
The concept of structure learning in action. ***A***, Unconstrained action space. Before experiencing perturbations, the action space is unbiased. ***B***, Action space constrained by archery practice. With experience, an archer will learn the general principle that she should aim in the opposite direction and with sufficient magnitude to counter an array of wind velocities. Thus, the action space should be constrained by azimuthal changes in aim. ***C***, Action space constrained by rotations. Likewise, when participants experience rotational perturbations, they learn to exploit the off-diagonal terms of the rotation matrix. Thus, the action space should be constrained by searches along a ring.

Imagine a novice archer attempting to hit a bullseye on a windy day. Initially, she may not know which set of actions to take to counter the crosswind–whether she should aim side-to-side or up-and-down (Fig. 1*A*)–but with practice she will learn to aim in the opposite direction and with sufficient magnitude to counter the wind (Fig. 1*B*). From this experience, she can also extract a general principle: she should always aim in the direction opposite to the wind. Her learning rate on future windy days will be dramatically faster because she no longer must search the entire space of potential actions.

This example reflects a form of structural learning in action: the ability to speed learning for novel, yet isostructural tasks by abstracting covariances from sensory inputs to constrain the space of potential solutions (Braun et al., 2010). Indeed, structural learning has been shown to afford faster learning in a visuomotor adaptation task (Braun et al., 2009), which induces an angular mismatch between hand and cursor movements (Krakauer, 2009). To probe structural learning, Braun et al. (2009) trained participants to overcome rotations that changed in direction and magnitude. Critically, they changed the rotation every eight trials and drew each rotation from a zero-mean distribution to prevent learning accumulation. Following this training phase, participants were exposed to a novel, consistent rotation. These participants were faster to counter this rotation relative to a control group that never experienced a perturbation and a “random” group exposed to a set of combined perturbations.

From a computational perspective, this benefit may arise from the identification of the covariance structure of task parameters, which constrains the dimensionality of the hypothesis space and consequently speeds the search for a solution. Consider the transformation matrix in Equation 1, which relates cursor movements to hand movements. The goal of learning is to fully parameterize the matrix (*a*, *b*, *c*, *d*), but the structural learning perturbation schedule prevents this because the rotation direction and magnitude change throughout training, overwriting the matrix parameters. Instead, structural learning exploits the relationship between the off-diagonal terms of the rotation transformation matrix (Eq. 2). The abstraction of this relationship (Eq. 3) collapses the dimensionality of the search space, speeding the acquisition of the parametric relationship between hand and cursor movements within the trained class, which affords faster learning (Fig. 1*C*).

(1) $$\begin{array}{l} \left[\begin{array}{c} x_{cursor}\\ y_{cursor} \end{array}\right]=\left[\begin{array}{cc} a & b\\ c & d \end{array}\right]\left[\begin{array}{c} x_{hand}\\ y_{hand} \end{array}\right] \end{array}$$

(2) $$\begin{array}{l} \left[\begin{array}{c} x_{cursor}\\ y_{curs\text{o}r} \end{array}\right]=\left[\begin{array}{cc} \text{cos}(\theta_{rotation}) & \sin(\theta_{rotation})\\ -\sin(\theta_{rotation}) & \cos(\theta_{rotation}) \end{array}\right]\left[\begin{array}{c} x_{hand}\\ y_{hand} \end{array}\right] \end{array}$$

(3) $$\begin{array}{l} \left[\begin{array}{c} x_{cursor}\\ y_{cursor} \end{array}\right]=\left[\begin{array}{cc} a & b\\ -b & a \end{array}\right]\left[\begin{array}{c} x_{hand}\\ y_{hand} \end{array}\right] \end{array}$$

This abstraction is presumed to be implicit (Genewein et al., 2015) and has been represented within an optimal feedback control framework as the result of an adaptive internal model (Braun et al., 2010). However, this interpretation stands in contrast to a recent series of findings demonstrating that explicitly accessible re-aiming processes constitute the majority of learning (Heuer and Hegele, 2008; Hegele and Heuer, 2010; Taylor et al., 2014; Bond and Taylor, 2015; McDougle et al., 2015; Brudner et al., 2016; Day et al., 2016; Poh et al., 2016). We previously found that explicit re-aiming composed the flexible component of performance across a range of rotation magnitudes while implicit recalibration exhibited a stereotyped response (Bond and Taylor, 2015). Furthermore, Morehead and colleagues showed that savings, a related phenomenon, was entirely the result of explicit re-aiming (Morehead et al., 2015). Altogether, there is ample motivation to further investigate whether structural learning can be expressed at an explicit level.

**Figure 2.**
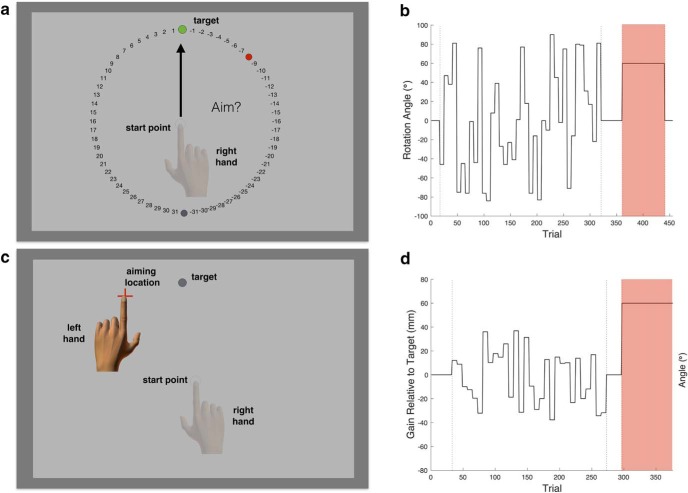
Reporting methods and variable perturbation schedules. ***A***, In experiment 1, participants reported their aim using a circular array of numbered landmarks which rotated with the target location such that the numbers 1 and −1 were adjacent to all target locations. ***B***, Perturbation schedule. The exposure phase trained participants on the rotation structure using a zero-mean rotation sequence drawn from a uniform distribution. In the highlighted test phase, all participants experienced a novel rotation of 60°. ***C***, In experiment 2, participants reported their intended reach endpoint by tapping a touch screen with the left hand. A red crosshair marked the tapped location and participants could tap anywhere on the screen. ***D***, Incongruent perturbation schedule. The basic experimental design for experiment 2 largely reflects that of experiment 1. Participants in the gain group experienced radial perturbations during the exposure phase, which were incongruent with the structure of the test perturbation (60° rotation). For the gain group, all phases except the test phase show the radial perturbation relative to the target, such that negative values indicate a negative scaling of cursor feedback and positive values indicate a positive scaling of cursor feedback (left *y*-axis). Because the test phase perturbation is a rotation, the perturbation for that phase is plotted as an angle (right *y*-axis).

In experiment 1, we tested whether explicit re-aiming could contribute to the phenomenon of structural learning by combining a recently developed technique to measure re-aiming behavior with the structural learning perturbation schedule from Braun et al. (2009; Fig. 2). We found that a variable rotation schedule drastically improved the learning rate for a novel rotation and that explicit re-aiming was entirely responsible for this effect. In experiment 2, we investigated whether re-aiming during the test phase was sensitive to the trained perturbation structure or more consistent with a generalized heuristic. We discovered that participants only showed learning rate benefits when training and test phase perturbations were drawn from the same structure, suggesting that rotation structure learning is accomplished via structure specific re-aiming.

## Materials and Methods

### Participants

Eighty-two participants (18.1–22.8 years, 39 female) were recruited from the research subject pool maintained by the Psychology Department at Princeton University or from the local community. One participant was excluded for failure to follow task instructions. Each participant received either course credit or $12 for participation. All participants were right-handed, verified using the Edinburgh Handedness Inventory (Oldfield, 1971), and reported normal or corrected-to-normal vision. Our research protocol was approved by the Princeton University Institutional Review Board and each participant gave informed consent before participation.

### Experiment 1 procedures

Before beginning each trial, the participant was required to position their hand at the center of a digitizing tablet while holding a digitizing pen (Intuos 3, Wacom). The tablet sampled movement trajectories at 100 Hz. Participants were capable of moving anywhere within the tablet active space (measuring 32.5 × 20.3 cm). Visual feedback was presented by a 43.18 cm, 1024 × 768 pixel, 60 Hz LCD monitor (Dell) that was horizontally mounted 24 cm above the tablet, occluding vision of the limb. To aid participants in finding the center of the tablet quickly, a circle either expanded or contracted with the radial distance of the participant’s hand position from the center of the tablet. Once the participant’s hand was 6 mm from the center of the start position (diameter, ⌀ = 5 mm), a white circular cursor (⌀ = 4 mm) appeared. After maintaining the start position for 1 s, a green circular target appeared (⌀ = 7 mm) at one of four target locations (cardinal axes: 0°:90°:270°) along a virtual ring with a radius of 9 cm. Each target location was pseudorandomly selected such that no target location repeated within an epoch of four trials and each participant received a different sequence of targets.

Participants were instructed to make a fast “shooting” movement toward the target location. Cursor feedback was provided throughout the reach and once the participant’s hand position exceeded 9 cm from the start point, the cursor turned red and its position was frozen, remaining on-screen for 1.5 s. If the movement duration exceeded 0.4 s, participants received an auditory warning (“too slow”) to encourage ballistic reaching movements. If the cursor position overlapped the target position, a pleasant chime sounded and the participant was awarded one point; otherwise, a harsh buzz played and zero points were awarded. Participants received a 5-s reminder of their absolute score and the proportion of points awarded after each 40 trial interval. The experiment was controlled by custom software written in Python (http://python.org) running on a laptop computer (Macbook Pro, Apple).

For certain phases of the experiment (see the final paragraph of this section), the visual workspace also included a virtual ring of numbers ranging from 1 to 31 and −1 to −31, with each number spaced 5.625° apart (Fig. 2*A*). These numbered landmarks rotated with the target position such that if a target were presented at a 90° angle (straight ahead), the number 1 would be presented at 95.625° and the number −1 would be presented at 84.375° (relative to the positive horizontal axis). Directly before the beginning of the aiming section of the baseline phase, participants were instructed: “You may have noticed that there were little numbers flanking the target. I would like you to tell me, before moving, the number that you think you should aim toward to get the cursor on the target. So if you think that you should aim directly at the target, then please say ‘green.’ But if you think that you should aim somewhere else to get the cursor on the target, please tell me what that number is.”

If a participant failed to report their aim, the experimenter reminded the participant to please continue to report the number to which they were aiming before moving. The experimenter coded the missed report for such trials as not-a-number (NaN), which accounted for 0.23% of trials.

Experiment 1 conformed to the following block format. First, participants made direct reaching movements to the targets with online cursor feedback to become familiarized with the basic task (first half of baseline phase: eight trials). Then, consistent with our factorial design (see final paragraph of this section), half of the participants were trained to verbally report their aiming location using the numbered landmarks on the screen (Fig. 2*A*) before moving on each trial (second half of baseline phase: eight trials). Next, also according to our factorial design, half of the participants were exposed to a pseudorandom perturbation schedule which consisted of rotations that varied in direction and magnitude (exposure phase: 304 trials). Each participant received a unique perturbation schedule.

Following the procedure used by Braun et al. (2009), a particular rotation was experienced for eight trials before changing to a new, pseudorandomly selected rotation. The rotations were drawn from a uniform distribution ranging from −90 to 90°, excluding 0°, and were chosen to have a zero mean across the exposure phase to prevent the accumulation of learning (Davidson and Wolpert, 2003). We also excluded rotation sizes within 10° of the test phase rotation (60°) and its inverse (−60°). We excluded these rotation values to isolate our measure of structural learning from visuomotor savings. Figure 2*B* illustrates an example perturbation schedule during the exposure phase. To washout the potential effect of any learned bias during the exposure phase, veridical feedback was restored (feedback-washout phase*:* 40 trials). Following this phase, participants experienced a counterclockwise 60° rotation (test phase: 80 trials). Finally, to measure aftereffects, all cursor feedback was removed and participants were instructed to reach directly to the target (washout phase: 16 trials). If participants were asked to report their aiming, the virtual ring of numbers was also erased during the washout phase.

Forty participants were divided equally into four groups according to a 2 × 2 factorial design with rotation structure exposure (structure) and verbal reporting (report) as factors. We included report as a factor to determine if the reporting procedure biased structural learning. The structure-report group experienced pseudorandom rotations during the exposure phase and reported their aiming location throughout the baseline (second half), exposure, feedback-washout, and test phases. Participants in the nostructure-report group did not experience perturbations during the exposure phase, but they were instructed to report their aiming locations. The structure-noreport group experienced rotational structure during the exposure phase, but never reported their aiming locations and the virtual ring of numbers was absent from the workspace. Finally, the nostructure-noreport group did not experience structure or report their aiming location at any point during the experiment.

### Experiment 1 analyses

Statistical analysis and data visualization were conducted using custom scripts written in R (R Foundation for Statistical Computing, RRID:SCR_001905) and MATLAB (MathWorks, RRID:SCR_001622). Kinematic data and aiming data were transformed from Cartesian to polar coordinates and rotated to a common axis such that the target was positioned at 0° (directly to the right). We operationalized kinematic performance using endpoint hand angle, which measures the angle between the target and the endpoint of the reach trajectory. Positive angles indicate a counterclockwise deviation from the target and negative angles indicate a clockwise deviation from the target. We quantified explicit learning by multiplying the verbally reported landmark by the spacing of the numbered landmarks (5.625°) for each trial. Implicit learning was computed by subtracting aiming position from the endpoint hand angle for each trial.

To test for the presence of baseline differences in kinematic performance across groups, we submitted the average endpoint hand angles over the last eight trials (epoch) of the baseline phase to a two-way ANOVA with factors of structure and report. To examine how responsive participants were to the variable perturbation schedule, we cross-correlated endpoint hand angles with the exposure phase solution for each participant to find the lag between time series that maximized their correlation. All correlation coefficients are calculated using the optimal lag for a given participant. We used a maximum lag of eight trials to reflect the length of each perturbation epoch during the exposure phase. We report the median and interquartile range (IQR) of the optimal lag for each group and compare correlations between groups exposed to structure using a two-sample *t* test. For the group that reported their aim during structure training (the structure-report group), we also report the median lag and mean correlation for explicit re-aiming and implicit learning.

To quantify how accurately participants opposed the perturbation series, we regressed the endpoint hand angle on the solution, using the slope of the linear fit as a proxy for reach accuracy in the exposure phase. For the structure-report group, we also regressed explicit re-aiming and implicit learning on the solution. We assumed that the closer the slope coefficient was to a value of 1, the better the participant tracked the exposure phase solution. To determine if the slopes for a particular group were significantly different from zero, we conducted one-sample *t* tests. We conducted a two-sample *t* test to assess whether there were significant differences between endpoint-hand-angle-solution slopes for each group that experienced structural training (structure-report and structure-noreport).

To ensure that the feedback-washout phase removed any bias that could have been induced by the exposure phase, we submitted the endpoint hand angles in the last epoch of the feedback-washout phase to a two-way ANOVA with factors of structure and report. Likewise, for the reporting groups, we tested whether any aiming bias induced by the exposure phase was removed by conducting a two-sample *t* test on aiming angles associated with the last epoch of the feedback-washout phase.

Our key dependent measure was learning rate in the test phase. To determine if the reporting procedure affected structural learning, we submitted the average endpoint hand angles over the first eight trials, our proxy for learning rate, to a two-way ANOVA with factors of structure exposure and reporting. Next, we sought to determine if changes in endpoint hand angle are attributable to changes in explicit re-aiming processes or implicit learning. Because explicit and implicit learning values are correlated, we chose to conduct a multivariate ANOVA (MANOVA) with a single factor of structure exposure, using explicit learning and implicit learning values during the first epoch of the test phase as our dependent variables.

Finally, to quantify aftereffects, we first subtracted average endpoint hand angles during the last epoch of the baseline phase from the average endpoint hand angles over the first eight trials of the no-feedback-washout phase for each participant. This preprocessing step allowed us to remove the influence of kinematic bias from our assessment of aftereffects. We then submitted these baseline-subtracted endpoint hand angles to a two-way ANOVA with factors of reporting and structure exposure. Because forward model adaptation quickly deteriorates when feedback is absent (Kitago et al., 2013), we only used data collected during the first epoch of this phase.

Note that we chose to quantify learning and aftereffects as performance averaged over eight trials instead of fitting an exponential function because we know that explicit re-aiming is highly nonmonotonic (Taylor et al., 2014; Bond and Taylor 2015) and because we know that exponential functions may not be representative of individual learning curves (Gallistel et al., 2004). This approach is consistent with previous studies using a similar reporting technique (Taylor et al., 2014; Bond and Taylor, 2015; Anglin et al., 2017).

Except where noted, we describe data using the mean and standard deviation. We consider comparisons yielding *p* < 0.05 to be statistically significant and comparisons yielding *p* < 0.10 to be marginally significant. Superscript letters associated with analyses correspond to the statistical tests shown in Table 3.

### Experiment 2 procedures

Similar to experiment 1, participants performed center-out reaching movements by sliding a digitizing pen across a digitizing tablet. The distance to the target was decreased to 7 cm to accommodate gain perturbations. The visual display was presented by a 1024 × 768 pixel, 60 Hz, touchscreen-compatible monitor (Acer) mounted 23.5 cm above the tablet. At the start of a trial, participants used radial feedback to bring their hand to the starting location (⌀ = 6 mm). After keeping their hand at the start position for 0.5 s, a gray target (⌀ = 8 mm) was displayed 7 cm from the start position. The targets could appear in one of eight locations (0°:45°:315°) and were pseudorandomized such that no target location was repeated until all targets were visited.

To assess adaptation to gain perturbations, participants in experiment 2 were required to solve the radial and angular component of the task to terminate the cursor within the target region (i.e., “point-to-point” movements). This meant that if the cursor was unperturbed, then participants would need to reach to the target distance and the target angle for a successful trial. If the cursor was perturbed by a gain, then participants would need to oppose the radial component of the perturbation but also match the target angle to terminate the cursor within the target region. For a successful rotation trial, participants would need to oppose the angular component of the perturbation but also match the target distance. Note that because it was necessary to have participants perform point-to-point movements to accommodate gain perturbations, these movement requirements are different from the shooting movements used in experiment 1. Cursor feedback (⌀ = 5 mm) was removed at the start of the movement, which was defined as beginning once the hand was 9 mm from the start position. Feedback, in the form of cursor position, was restored at the end of the reach, which was defined as when the reach speed fell below 7 cm/s, and displayed for 1 s. If the cursor position overlapped the target position, the participant heard a pleasant chime and the target turned from gray to green. An unsuccessful trial was met with a buzz and the gray target turned blue. Then, the screen was erased and participants were required to find the start point using radial feedback to begin the next trial, as described above.

Another difference between experiments 1 and 2 concerned how participants reported their explicit aiming location. In experiment 2, participants were asked to indicate where they planned to move to terminate their cursor within the target by tapping an intended reach endpoint on a touchscreen monitor using their left hand (Fig. 2*C*). Importantly, because reporting in this experiment was unconstrained, this measurement of explicit aiming yielded higher resolution data than the verbal reporting method in experiment 1. Additionally, the absence of numbers to demarcate potential reporting locations allowed for a less cued assessment of aiming behavior. After the participant tapped the screen, a red crosshair marked the tapped location and remained on-screen for 1 s. Participants then rested their left hand on the table, away from the visual workspace. Additionally, while experiment 2 followed the same blocked schedule as in experiment 1, experiment 2 deviated from the trial sequence in experiment 1 in two ways. First, because touchscreen reporting takes more practice, the number of trials in the baseline phase was increased from 16 to 32. Second, because touchscreen reporting increases inter-trial time, the length of the exposure phase was decreased from 304 to 240 trials.

Immediately before the onset of the first aiming trial, the experimenter gave the following instructions: “So far, the cursor has followed your hand position. At some point in the experiment, we may manipulate the relationship between your movement and the cursor. Therefore, a direct aim to the target may not be effective. You may need to aim to another location to get the cursor on the target. So, I’d like you to tap the screen wherever you think that you should move your hand to get the cursor on the target. For example, if you think that you should move your hand directly underneath the target to get your cursor to hit the target, then touch the target. If you think that you should move your hand anywhere else to get the cursor on the target, then touch that spot.”

Additionally, participants were encouraged to ask questions if they found the instructions to be unclear.

Forty-two participants were equally divided into three groups to examine how exposure to different perturbation structures affected acquisition of a new perturbation from the same or different structure. We exposed participants to either rotation or gain perturbations (Fig. 2*D*) or, in the control group, veridical feedback during the exposure phase before they experienced a rotation perturbation in the test phase. As in experiment 1, each participant received a unique perturbation schedule.

The rotation group experienced rotational perturbations during the exposure phase before being exposed to a 60° rotation in the test phase (congruent schedule). These rotational perturbations were drawn from a uniform distribution of integers ranging from −90° to 90°, excluding 0° and values ±10° of the rotation value in the test phase (60°) and its inverse, −60°. The mean value of selected exposure phase rotation perturbations for any given subject was 0° (μ exposure phase maximum rotation across subjects = 86.64°, σ = 3.48°; μ exposure phase minimum rotation across subjects = −87.29°, σ = 2.7°).

The gain group experienced a sequence of radial perturbations during the exposure phase before a 60° rotation in the test phase (incongruent schedule). Gain perturbations were drawn from a uniform distribution with a lower bound of 0.66 and an upper bound of 2.30, excluding 1. These parameters were chosen so that participants could successfully reach all target locations (the tablet size precluded using negative gains <0.66 because the reach solution would exceed the boundaries of the active tablet space). Because the size of the active tablet space did not allow for as broad a range of negative gains as positive gains and because of our constraint that each perturbation within a given participant’s exposure phase be unique, the mean value of selected exposure phase gain perturbations for each participant was not exactly 1 but biased toward a positive gain, with a modest tolerance for mean exposure phase values ranging from 0.90 to 1.10 (μ of exposure phase gain sequences across subjects: 1.04, σ = 0.05; μ exposure phase maximum gain across subjects = 1.98, σ = 0.17; μ exposure phase minimum gain across subjects = 0.66, σ = 0.01).

Finally, the control group did not experience any perturbation during the exposure phase, but experienced a 60° rotation in the test phase.

### Experiment 2 analyses

Reach trajectories were transformed into polar coordinates as in experiment 1. We quantified explicit learning as the x-y coordinates of the tapped aiming location, which were transformed into polar coordinates and rotated to a common axis. Our analyses for each phase of interest were similar to experiment 1, except that we performed one-way ANOVAs with a single factor of group (rotation, gain, control) in place of two-way ANOVAs. We did not seek to compare implicit learning between groups during the exposure phase as the perturbations were fundamentally different (rotations vs gains). However, we did analyze implicit learning during the first epoch of the test phase using a one-way ANOVA with a single factor of group. Additionally, to test whether the gain group showed persistent radial differences from the rotation and control groups during early test phase learning, we conducted two one-way ANOVAs on the first epoch of test phase reaching and aiming radii with a single factor of group. To test for dependence between explicit re-aiming and overall reaching in the early test phase, we conduct paired *t* tests between aiming and reaching values during the first epoch of the test phase for each group and correlate the explicit re-aiming and reaching distributions within a group. We follow the same conventions for statistical significance as in experiment 1. Superscript letters associated with analyses correspond to the statistical tests shown in Table 3.

### Power analysis

Because estimates of mean and variance were not available from Braun et al. (2009), we based our sample size (*N* = 10/group) on a prior sensorimotor adaptation task measuring aiming and using multiple rotation sizes. For experiment 2, however, we computed the sample size required to achieve similar effect sizes using learning rates (first eight-trial epoch in test phase) from the structure-report and nostructure-report groups from experiment 1. We focused on learning rate since our primary interest was in how structure in the exposure phase affected learning rate in the test phase. For the learning rate differences between structure-report and nostructure-report, the effect size as measured by Cohen’s f was 1.03 (structure-report: μ = −51.54°, σ = 21.33°; nostructure-report: μ = 7.63°, σ = 10.97°). Using a conservative α value of 0.01, we estimated that a sample size of 14 participants per group provided ample power.

## Results

### Experiment 1. Does structural learning arise from explicit re-aiming or implicit learning?

In experiment 1, we tested whether structural learning was expressed through explicit re-aiming or an implicit recalibration process.

#### Baseline phase

All participants practiced reaching to the target with veridical feedback to become familiarized with the task. In the second half of the baseline phase, participants practiced reaching to the target with veridical feedback while verbally reporting their intended aiming location using the virtual ring of numbers on-screen. To assess whether there were any baseline differences between groups that could affect exposure phase learning, we compared reaching performance (endpoint hand angles) across groups. Endpoint hand angles during the last epoch of the baseline phase were submitted to a two-way ANOVA with factors of structure and report (Table 1), which revealed no effect of structure (*F*_(1,36)_ = 0.60, *p* = 0.4441), a marginal effect of report (*F*_(1,36)_ = 4.12, *p* = 0.0498), and no interaction (*F*_(1,36)_ = 0.20, *p* = 0.6579)^a^. Because none of the participants had yet experienced a perturbation, we did not expect structure to modulate performance or interact with reporting. The marginal effect of reporting decreased baseline reach accuracy (Table 1, baseline section), but the magnitude of the maximum difference between reporting and nonreporting group averages was small, on the order of 3° (Table 1).

**Table 1.** Average endpoint hand angles, aiming angles, and estimates of implicit learning for each consistent experiment phase (excluding the exposure phase)

Experiment phase	Structure-report	Structure-noreport	Nostructure-report	Nostructure-noreport
Baseline				
Hand angle	2.98 ± 6.36	0.27 ± 1.44	1.64 ± 2.11	−0.09 ± 1.05
Aim	0 ± 0	—	0.14 ± 0.44	—
Implicit	0.76 ± 0.97	—	1.5 ± 2.13	—
Feedback washout				
Hand angle	−0.06 ± 1.38	1.74 ± 1.73	0.04 ± 0.75	−0.16 ± 0.82
Aim	−0.42 ± 0.95	—	0 ± 0	—
Implicit	3.64 ± 8.53	—	−0.12 ± 1.35	—
Early test				
Hand angle	−50.2 ± 5.70	−38.7 ± 21.91	−6.75 ± 21.07	−12.97 ± 19.06
Aim	−51.54 ± 5.33	—	−6.5 ± 19.96	—
Implicit	1.34 ± 4.23	—	−1.49 ± 20.94	—
No-feedback-washout				
Hand angle	4.91 ± 6.90	−2.89 ± 2.5	−6.45 ± 2.88	−6.44 ± 3.29

#### Exposure phase

To expose participants to rotational structure, structure-report and structure-noreport groups experienced a series of rotations pseudorandomly drawn from a zero-mean, uniform distribution. Note that our analyses of the exposure phase only focus on the groups that experienced structure. The groups that did not experience structure either continued to have similar or improved performance compared to the baseline phase (nostructure-noreport: *t*_(9)_ = −0.77, *p* = 0.4593; nostructure-report: *t*_(9)_ = 2.75, *p* = 0.0225; Table 1)^b^.

To examine how well participants in the structure groups tracked the variable perturbation schedule, we cross-correlated and regressed the endpoint hand angles with the exposure phase solution for each participant. We found that participants exposed to rotation structure quickly updated their movement vector during the exposure phase. The correlation coefficient between the hand angle and rotation solution was 0.83 ± 0.16 and 0.58 ± 0.21 for the structure-report and structure-noreport groups, respectively. The median of the optimal cross-correlation lag was 1 for the structure-report (IQR: 0) and structure-noreport groups (IQR: 1). Correlation coefficients between groups were significantly different (*t*_(18)_ = 2.94, *p* = 0.0088)^c^, indicating that reporting may have helped participants respond to the rotation sequence.

**Figure 3.**
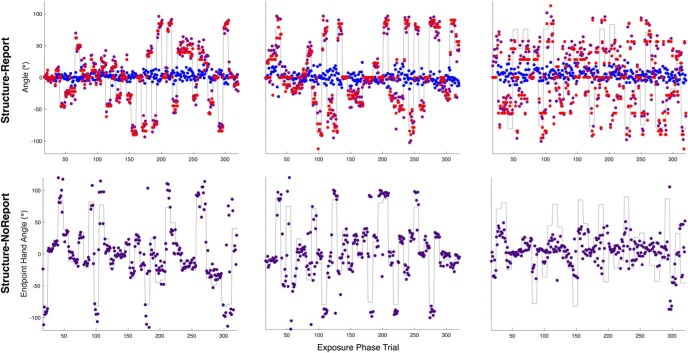
Experiment 1. Example reach performance. Endpoint hand angle (purple), for the best (first column), median (second column), and worst (third column) participants based on the slope of a linear regression of exposure phase endpoint hand angles on the rotation solution (gray lines). Note that the solution angle is simply the opposite of the rotation angle. Top row, Explicit re-aiming (red) and implicit learning (blue) for participants in the structure-report group. Bottom row, Endpoint hand angle for participants in the structure-noreport group.

Likewise, the average slopes for the structure-report and structure-noreport groups were 0.72 ± 0.15 and 0.52 ± 0.22, respectively. The distribution of slopes within each group was significantly different from zero (structure-report: *t*_(9)_ = 14.90, *p* = 1.1967e-07; structure-noreport: *t*_(9)_ = 7.40, *p* = 4.0938e-05)^d^ and there was a significant difference between groups (*t*_(18)_ = 2.45, *p* = 0.0249)^e^. Taken together, these analyses suggest that both groups learned to counter the pseudorandom visuomotor rotations during the exposure phase, but the act of reporting may have augmented performance. Note that because the sequence of rotations was different for each participant, we cannot plot a subject-averaged time series of exposure phase performance. Instead, Figure 3 shows performance from a range of participants in the structure-report and structure-noreport groups.

For the structure-report group, we also cross-correlated aiming angles and our estimate of implicit learning with the exposure phase solution. As shown in the sample time courses (Fig. 3), explicit learning was highly responsive to the perturbation series. Indeed, we found that reported aiming and movement vectors were updated simultaneously, with a correlation coefficient of 0.84 ± 0.17 and a median optimal lag of 1 (IQR: 0), strikingly, these aiming lag values were exactly those calculated for hand angles, further reinforcing their synchronous relationship. The average explicit learning slope was 0.73 ± 0.17 (*t*_(9)_ = 13.31, *p* = 3.1703e-07)^f^, suggesting that explicit re-aiming accounted for the majority of learning during the exposure phase. In contrast, when we performed the same analyses on the implicit component of learning, we found that the correlation coefficient was only 0.13 ± 0.05 and with a median lag of 4 and high variability among subjects (IQR: 7). The average implicit learning slope was shallow (0.00 ± 0.06) and the distribution of implicit learning slopes was not significantly different from zero (*t*_(9)_ = −0.12, *p* = 0.9106)^g^. These results are not entirely unexpected because recent research has shown that re-aiming underlies quick performance improvement and because the exposure phase perturbation schedule was designed to minimize the contribution of implicit learning.

#### Feedback-washout phase

Directly after the exposure phase, all participants were exposed to veridical feedback to ensure that any bias induced by the exposure phase was removed before the test phase. To confirm that movements were unbiased by the perturbation series during the last epoch of the feedback-washout phase, we conducted a two-way ANOVA with factors of structure and report. There was a marginal effect of reporting (*F*_(1,36)_ = 4.17, *p* = 0.0484), an effect of structure (*F*_(1,36)_ = 5.31, *p* = 0.0271), and an interaction between reporting and structure (*F*_(1,36)_ = 6.54, *p* = 0.0149)^h^, indicating that reporting modulated the influence of structure on hand angles. *Post hoc*, Bonferroni-corrected *t* tests between groups indicated that there was a difference between the structure-report group and the structure-noreport group (*p* = 0.015) but no difference between the structure-report group and the nostructure-report and nostructure-noreport groups (*p* = 0.99 for both comparisons). The structure-noreport group was different from the nostructure-report group (*p* = 0.024) and the nostructure-noreport group (*p* = 0.009). There was no difference between the nostructure-report group and the nostructure-noreport group (*p* = 0.99). Overall, the effect of reporting and structure exposure on endpoint hand angles was inconsistent, and when present, affected reaching to a minor degree. The magnitude of the maximum difference between group averages was small, ∼2° (Table 1).

There was no difference between aiming angles during the last epoch of the feedback-washout phase for the structure-report and nostructure-report groups (*t*_(18)_ = −1.41, *p* = 0.1769)^i^. These results indicate that the aiming behavior induced by the exposure phase was washed out before the test phase, and while there were differences between group endpoint hand angles, these differences were minor.

#### Test phase

In the test phase, all participants were exposed to a 60° counterclockwise rotation. Because a change in learning rate is the signature of structural learning, learning rate was our primary dependent measure in the test phase. Based on prior work, we predicted that the groups exposed to rotation structure would have a greater learning rate compared to groups that were not exposed to structure. Two open questions remain: does the reporting procedure affect structural learning and does the increased learning rate arise from explicit re-aiming or implicit learning?

**Figure 4.**
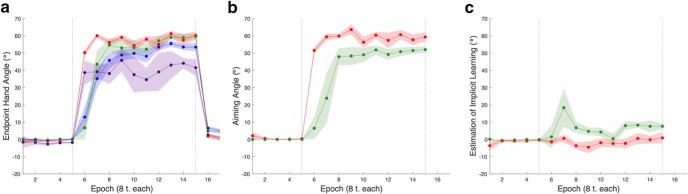
Experiment 1. The feedback-washout phase and the time course of learning during the test phase. ***A***, Overall learning. Overall learning is accelerated in the groups exposed to rotation structure (structure-report, shown in red, and structure-noreport, shown in purple) relative to groups without structure exposure (nostructure-report, shown in forest green, and nostructure-noreport, shown in dark blue). ***B***, Explicit re-aiming. Explicit re-aiming composes all of performance in the structure-report group and the majority of performance in the nostructure-report group. ***C***, Implicit learning. Implicit learning in the reporting groups, structure-report and nostructure-report. Error is shown as SEM.

To address the first question, we submitted the average endpoint hand angles over the first eight trials, our proxy for learning rate, to a two-way ANOVA with factors of structure exposure and reporting. We found a main effect of structure (*F*_(1,36)_ = 36.28, *p* = 6.48e-07), no effect of reporting (*F*_(1,36)_ = 0.21, *p* = 0.648), and no interaction (*F*_(1,36)_ = 2.38, *p* = 0.132)^j^, indicating that the increase in learning rate was a consequence of rotation structure exposure rather than being cued to report an explicit re-aiming strategy and that reporting did not modulate the effect of structural exposure on learning rates (Fig. 4*A*; Table 1). Note that these results do not provide evidence to suggest that explicit re-aiming processes do not express structural learning, but that probing this component of learning does not significantly affect learning rate.

We wanted to determine if the increase in learning rate evident in endpoint hand angle was attributable to changes in explicit re-aiming processes or implicit learning. Because explicit re-aiming and our estimate of implicit learning are correlated, we submitted explicit learning and implicit learning values during the first epoch of the test phase to a MANOVA with a single factor of structure exposure. There was a main effect of structure exposure on performance (*F*_(1,18)_ = 24.60, *p* = 9.582e-06, Pillai’s trace = 0.74)^k^. Explicit re-aiming differed with structure exposure (*F*_(1,18)_ = 47.54, *p* = 1.901e-06) while implicit learning did not (*F*_(1,18)_ = 0.18, *p* = 0.6803; see Table 1 for average explicit learning and implicit learning values). Altogether, these results indicate that differences in learning rate for a novel rotation are attributable to changes in explicit re-aiming, not implicit learning (Fig. 4*B*,*C*).

#### Aftereffects

During the no-feedback-washout phase, the aiming landmarks were removed and participants were instructed to reach directly to the target to measure the implicit aftereffects of learning in the test phase. The averaged, baseline-subtracted endpoint hand angles in the first epoch of the no-feedback-washout phase were submitted to a two-way ANOVA with factors of report and structure. We found no effect of reporting on aftereffect size (*F*_(1,36)_ = 0.57, *p* = 0.4541)^l^. However, there was an unexpected, albeit marginal, effect of structure exposure (*F*_(1,36)_ = 3.55, *p* = 0.0677)^l^, suggesting that exposure to pseudorandomly varying rotations suppresses the measured aftereffect size for a novel rotation (Table 1). There was no interaction between reporting and structure exposure (*F*_(1,36)_ = 0.55, *p* = 0.4620)^l^, indicating that reporting did not modulate the effect of structural learning on aftereffects.

### Experiment 2. Is structural learning specific to the trained perturbation structure or expressed via a general aiming heuristic?

In experiment 2, we tested the specificity of the training needed to increase the learning rate for a novel rotation. In contrast to experiment 1, we exposed participants to either rotation perturbations or gain perturbations, such that the training structure was either consistent or inconsistent with the rotation structure in the test phase.

#### Baseline phase

All participants practiced reaching to the target with veridical endpoint feedback to become familiarized with the task. In the second half of the baseline phase, participants practiced reaching to the target while tapping a touchscreen to report their intended reach endpoint (Fig. 2*C*). To assess whether there were any baseline differences between groups that could affect exposure phase learning, we compared reaching performance. There were no differences across groups in the angular component of reaching (*F*_(2,39)_ = 0.76, *p* = 0.4764)^m^ during the last epoch of the baseline phase. There was, however, a significant difference between groups for baseline reach distances (*F*_(2,39)_ = 4.44, *p* = 0.0182)^n^. *Post hoc*, Bonferroni-corrected pairwise comparisons revealed a significant difference between the rotation and gain group reach distances (*p* = 0.0278), a marginally significant difference between gain and control group reach distances (*p* = 0.0651), and no significant difference between rotation and control group reach distances (*p* = 0.99). The magnitude of the difference between group means was minor, measuring 6.55 mm at maximum (Table 2).

**Table 2.** Average endpoint hand angles/radii and aiming angles/radii for each consistent experiment phase

*Experiment phase*	*Rotation group*	*Gain group*	*Control group*
Baseline			
Hand angle/radius	3.71 ± 2.47/69.24 ± 6.20	5.52 ± 6.20/62.68 ± 6.35	4.20 ± 2.03/68.41 ± 6.44
Aim angle/radius	0.77 ± 0.51/71.60 ± 2.42	0.42 ± 0.0.86/71.21 ± 1.64	1.19 ± 1.28/70.84 ± 2.89
Feedback-washout			
Hand angle/radius	4.35 ± 4.19/67.64 ± 4.46	2.91 ± 2.75/74.16 ± 6.93	2.55 ± 2.09/70.84 ± 3.38
Aim angle/radius	−0.48 ± 2.20/72.45 ± 6.19	0.18 ± 1.20/76.19 ± 11.91	−0.49 ± 1.63/73.38 ± 4.65
Test			
Hand angle/radius	−42.53 ± 11.21/71.91 ± 6.43	−9.81 ± 23.48/73.27 ± 11.34	−14.83 ± 13.14/73.08 ± 6.14
Aim angle/radius	−43.23 ± 11.63/73.32 ± 4.75	−5.59 ± 34.19/72.42 ± 13.62	−16.65 ± 11.71/74.42 ± 7.73

Error is shown as SD. Angles are measured in degrees and radii are measured in millimeters.

#### Exposure phase

To determine if structural learning was specific to the form of the trained perturbation structure, we exposed the gain group to gain perturbations (Fig. 2*D*) and the rotation group to rotation perturbations during the exposure phase. To prevent participants from transferring an average representation of the perturbation series, we ensured that the perturbations were drawn from a uniform distribution such that the rotation series averaged to zero and the gain series averaged to approximately one for any given participant. The control group continued to experience veridical feedback during this phase, which improved performance such that participants more closely approximated hitting the target (*t*_(13)_ = 2.64, *p* = 0.0203)^o^.

**Figure 5.**
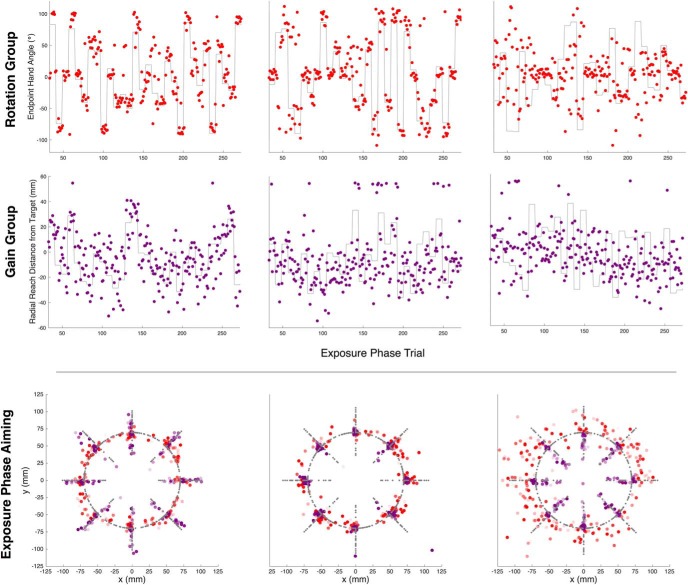
Experiment 2. The best (first column), median (second column), and worst (third column) performance based on the slope of a linear regression of exposure phase reach performance on the perturbation solution (gray lines). Top row, Endpoint hand angle for the rotation group (red). Second row, Radial distance of reach endpoint relative to the target distance for the gain group (purple). Negative values indicate a reach distance greater than the target distance and positive values indicate a reach distance shorter than the target distance. Bottom row, Exposure phase aiming locations in *x-y* space. Exposure phase aiming from sample subject from the gain (shown in purple) and rotation (shown in red) groups. α value scales with trial number such that the last trial within an epoch is most opaque. The gray points represent the solution for a given trial. Note that a sample subject is not shown from the control group because they simply received veridical feedback.

To examine how well participants in the rotation group opposed the variable perturbation schedule, we cross-correlated and regressed endpoint hand angles with the exposure phase solution for each participant. We found that participants in the rotation group quickly updated their movement vectors in response to the perturbation sequence. The median lag which maximized the correlation between reaching and solution time courses for participants in the rotation group was 1 (IQR: 1), and the mean correlation between endpoint hand angles and the solution was 0.59 ± 0.24. Because we perturbed the radial component of movement for the gain group, we conducted the cross-correlation and regression analyses of performance in that group using endpoint hand radii. The median lag for the gain group was 2 (IQR: 2) and the mean correlation between endpoint hand radii and the solution was 0.42 ± 0.16. Correlation coefficients between groups were marginally different (*t*_(26)_ = −2.10, *p* = 0.0456)^p^, suggesting that participants may be more sensitive to perturbations affecting the angular component of feedback. Despite this difference in sensitivity to perturbation types, participants were capable of tracking both radial and angular perturbations (see Figure 5 for exposure phase performance in sample gain and rotation participants).

For the rotation group, the average slope between the exposure phase hand angle and the rotation solution was 0.51 ± 0.24 and the distribution of rotation slopes was significantly different from zero (*t*_(13)_ = 8.09, *p* = 1.9788e-06)^q^. The average slope for the gain group was 0.33 ± 0.18 and the distribution of gain slopes was significantly different from zero (*t*_(13)_ = 6.88, *p* = 1.1109e-05)^q^. Rotation slopes were significantly greater than gain slopes (*t*_(26)_ = −2.20, *p* = 0.0365)^r^, providing further support for the idea that participants more accurately track rotational perturbations than gain perturbations.

#### Feedback-washout phase

The purpose of the feedback-washout phase was to use veridical feedback to remove any influence that the exposure phase may have had on participants’ movements. To confirm that movements were unbiased by the perturbation series during the last epoch of the feedback-washout phase, we conducted four one-way ANOVAs with a single factor of group, comparing angular and radial components of aiming and reach performance in the last epoch of the feedback-washout phase. There were no differences in endpoint hand angles (*F*_(2,39)_ = 1.3, *p* = 0.2850)^s^ or aiming angles (*F*_(2,39)_ = 0.68, *p* = 0.5109)^t^ between groups. However, there was a significant difference in endpoint hand radii between groups (*F*_(2,39)_ = 5.63, *p* = 0.0071)^u^, but the maximum difference between mean group radii was small, measuring ∼6.52 mm (rotation-gain: *p* = 0.0053, all other comparisons insignificant; Table 2), which was similar to the difference observed in the baseline phase. There were no between-group differences in aiming radii (*F*_(2,39)_ = 0.79, *p* = 0.4608)^v^.

#### Test phase

In the test phase, all participants were exposed to a 60° counterclockwise rotation. Our primary question for this experiment was: does structural exposure have a structure-specific effect on learning? We predicted that if the exposure phase simply taught participants to use a general aiming heuristic, then gain and rotation groups might have similar test phase performance and both groups would learn more quickly than the control group. However, if participants learned the perturbation structure, then the rotation group would improve performance in the test phase much more quickly than either the gain or control group.

To shed light on this, we submitted endpoint hand angles averaged over the first epoch of the test phase to a one-way ANOVA with a single factor of group. We found a significant difference between groups (*F*_(2,39)_ = 15.36, *p* = 1.2049e-05)^w^. A Bonferroni-corrected pairwise comparison showed a significant difference between the rotation and gain groups (*p* = 2.3705e-05) and a significant difference between rotation and control groups (*p* = 2.7993e-04). There was no difference between gain and control groups (*p* = 0.99).

To test whether the gain group showed persistent radial differences from the rotation and control groups during early test phase learning, we conducted two one-way ANOVAs on the first epoch of test phase reaching and aiming radii with a single factor of group. We found no differences in reaching radii (*F*_(2,39)_ = 0.11, *p* = 0.8956)^x^ or aiming radii (*F*_(2,39)_ = 0.19, *p* = 0.8317)^y^ between groups, suggesting that differences in learning rate were restricted to the angular dimension (Table 2).

Based on our results from experiment 1, we predicted that explicit re-aiming drove this structure-specific effect on reach performance instead of implicit learning. To test this idea, we performed the same analysis as above using the reported aiming angles and our estimate of implicit learning during the first epoch of the test phase. Consistent with our prediction, we found a significant difference in re-aiming between groups (*F*_(2,39)_ = 10.90, *p* = 1.7326e-04)^z^ but no difference in implicit learning (*F*_(2,39)_ = 0.99, *p* = 0.3816)^aa^. A Bonferroni-corrected pairwise comparison of re-aiming revealed a significant difference between the rotation and gain groups (*p* = 1.5708e-04) and a significant difference between rotation and control groups (*p* = 0.0080). As above, there was no difference in re-aiming between gain and control groups (*p* = 0.5689).

**Figure 6.**
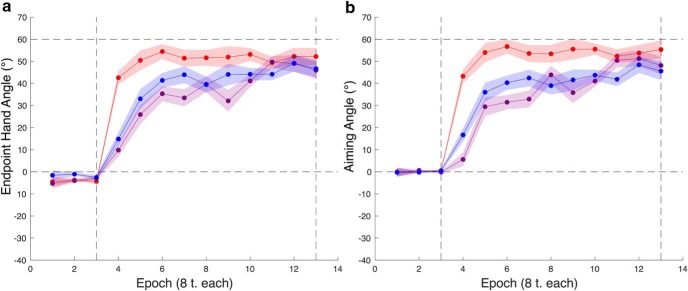
Experiment 2. The feedback-washout phase and the time course of test phase learning. ***A***, Overall learning. Overall learning is accelerated in the rotation group (shown in red) relative to both the gain (shown in purple) and control (shown in blue) groups. There is no difference between gain and control group learning rates. ***B***, Explicit re-aiming. Aiming patterns underlie overall performance, with the rotation group showing an explicit re-aiming learning rate commensurate with the overall learning rate. The same is true of the gain and control groups. Error is shown as SEM.

Learning rates for explicit re-aiming and reaching were indistinguishable for every group (paired *t* test; rotation: *t*_(13)_ = 0.35, *p* = 0.7356, gain: *t*_(13)_ = −0.84, *p* = 0.4160, control: *t*_(13)_ = 1.27, *p* = 0.2253)^bb^ and the distributions of explicit re-aiming and reaching learning rates were closely correlated for each group (rotation: *r* = 0.78, gain: *r* = 0.85, control: *r* = 0.91)^cc^. The synchronicity of re-aiming and movement vector updating is also clearly shown in the time courses of explicit re-aiming and reaching (Fig. 6).

Overall, these results favor the idea that exposure to perturbation structure leads to structure-specific effects on learning rate for a novel rotation. Consistent with our prediction, this increase in learning rate is mediated via explicit re-aiming.

## Discussion

In this study, we sought to shed light on whether explicit re-aiming could contribute to the phenomenon of structural learning. A prior study suggested that structural learning could not be attributable to an explicit, cognitive strategy because explicitly informing participants of the task solution did not improve performance (Genewein et al., 2015). However, the perturbation did not always follow the instructed strategy and, consequently, participants may not have trusted the strategy or applied it consistently. Furthermore, an instructed strategy can be worse for performance than self-discovery (Mazzoni and Krakauer, 2006; Gureckis and Markant, 2012) and in some cases may prevent the expression of learning (Reber, 1989).

To investigate whether structural learning can be expressed by an explicit process, we conducted two experiments, combining two techniques to assay explicit re-aiming behavior with the structural learning perturbation schedule from Braun et al. (2009). We found that prior experience with a variable rotation schedule drastically improved the learning rate for a novel rotation. This effect was entirely driven by explicit re-aiming. Additionally, participants only showed learning rate benefits when *exposure* and *test* phase perturbations were drawn from the same perturbation structure, suggesting that rotation structure learning is accomplished via structure specific re-aiming instead of a simple heuristic. Because the contribution of implicit learning was negligible, we suggest that the process responsible for structural learning in a sensorimotor adaptation task may be similar to those involved in other domains such as category learning (Ashby and Maddox, 2005; Huang-Pollock et al., 2011), concept learning (Goodman et al., 2008), and decision making (Frank et al., 2009).

### Structural learning of rotational perturbations is explicitly accessible

Our first experiment examined whether rotation metalearning was primarily expressed via explicit or implicit learning processes. Given the abundance of recent evidence to indicate that explicit learning underlies rapid changes in performance, we predicted that explicit learning would drive the increased learning rate in the groups which were exposed to rotation structure. Indeed, we found that explicit processes conferred the entirety of the learning rate benefit characteristic of a metalearning process.

Surprisingly, for participants who received rotation structure training, implicit learning and its corresponding aftereffects were smaller. Note that this was not an effect of reporting, as we found no difference between reporting and nonreporting groups. Indeed, test phase implicit learning in the nostructure-report group matched the degree of implicit learning found in a previous study (Bond and Taylor, 2015).

One possibility is that structure training indirectly affects implicit learning by changing explicit re-aiming processes. A recent study found that implicit generalization is centered about the aiming location and not the target, hand, or cursor position (Day et al., 2016). Thus, participants who aim to a greater magnitude will train implicit learning farther from the target location. If implicit learning is tied to an aiming location, then when participants are asked to stop aiming and instructed to reach directly to the target, as in the no-feedback washout phase of the current study, aftereffects will appear to be smaller. Correspondingly, if participants were instead asked to aim at their most frequent aiming location, aftereffects should be much greater (Day et al., 2016). In our study, it is likely that participants in the structure-report group more consistently aimed to a greater magnitude than the nostructure-report group. This would cause implicit learning to peak farther from the target location and become more localized. In contrast, the nostructure-report group may have more frequently aimed to locations between the target and the aiming solution, causing implicit learning to be tied to a wider spread of spatial positions, which could create the appearance of larger aftereffects in the nostructure-report group. While this simple explanation is attractive, it should be noted that implicit learning during the test phase also appeared to be different between groups, which cannot be fully accounted for by aiming-based generalization.

Regardless of the above possibilities, implicit learning does not appear to be capable of expressing structural learning. This implies that forward models, which are thought to underlie implicit learning in visuomotor adaptation tasks (Wolpert and Miall, 1996), are restricted to learning parametric, rather than structural, relationships between action and feedback. It is unlikely that the cerebellum, which has been consistently linked with performing computations akin to a forward model (Taylor et al., 2010; Izawa et al., 2012; Schlerf et al., 2012; Morehead et al., 2017), could facilitate structural learning. Instead, structural learning of rotations may rely on neural mechanisms common to explicit, rule-based systems in other domains, such as category learning (Ashby and Maddox, 2005; Huang-Pollock et al., 2011), concept learning (Goodman et al., 2008), and decision making (Frank et al., 2009). There is evidence to suggest that abstracting rules for action progressively activates the rostral-caudal axis (Badre et al., 2010), with increased activation in the prePMd as the search for relationships between action and feedback becomes more abstract. Given that the prefrontal cortex is consistently engaged in the early stages of learning a sensorimotor task (Shadmehr and Holcomb, 1997; Floyer-Lea and Matthews, 2004; Suzuki et al., 2004; Seidler et al., 2006; Anguera et al., 2007; Seidler et al., 2013) and patients with prefrontal lesions show impaired performance in these tasks (Slachevsky et al., 2001; Slachevsky et al., 2003; Taylor and Ivry, 2014; Taylor et al., 2014), perturbation structure learning tasks driven by explicit learning processes may also generate the same activation patterns during abstraction. However, forming abstractions in a larger space might tax the limit of explicit learning processes, and therefore such tasks might recruit the aid of multiple learning processes, including model-based and model-free reinforcement learning (Badre et al., 2010; Collins and Frank, 2016).

Alternatively, Herzfeld and colleagues suggested that the motor system changes its sensitivity to previously experienced errors, which could lead to savings or, perhaps, structural learning (Herzfeld et al., 2014). While a change in error sensitivity would be assumed to rely on implicit processes, it is entirely possible that this change in sensitivity is the result of an explicitly accessible re-aiming process. Future work is needed to dissociate the source of changes in error sensitivity using a paradigm similar to that of Herzfeld et al. (2014).

Finally, rotation magnitude may dictate whether structural learning is expressed through an explicit re-aiming or implicit learning process. Implicit learning appears to exhibit a highly stereotyped response (Bond and Taylor, 2015), and operates to a similar degree for any error between 7.5° and 90° (Morehead et al., 2017). In contrast, explicit re-aiming exhibits a dose-dependent response across a wide range of rotation magnitudes (Bond and Taylor, 2015). Even when rotations are small, explicit re-aiming reduces error during early learning while implicit learning accumulates (Bond and Taylor, 2015). However, the relative contribution of explicit and implicit processes has only been investigated for rotations ≥15°. Thus, it may be possible that participants do not explicitly reaim their movements for rotations smaller than 15°, and, as a consequence, structural learning may be expressed implicitly. Nevertheless, we think that this is an unlikely scenario given that implicit learning shows a highly stereotyped response (Morehead et al., 2017) and fails to exhibit savings (Morehead et al., 2015).

### Test phase learning rate improvements are driven by explicitly accessible structural learning, not heuristic aiming strategies

While our first experiment clearly demonstrated that explicit processes were responsible for an increased learning rate for a novel rotation, it failed to pinpoint the source of improved test phase performance because both simple aiming heuristics and rotation structure learning could yield the same benefit. In our second experiment, we investigated whether exposure to distinct perturbation structures resulted in structure-specific effects on learning a novel rotation. We predicted that if the exposure phase simply taught participants to use a general aiming heuristic, then gain and rotation test phase performance should be similar, with the control group exhibiting a decreased learning rate relative to these two groups. However, if participants learn perturbation structure, then the rotation group will learn much more quickly than either the gain or control group. We found that participants exposed to rotation structure during the exposure phase learned to counter a novel rotation much more quickly than participants exposed to either a veridical or gain structure. Remarkably, there was no difference in learning rate between groups exposed to either veridical or gain structure, demonstrating that the learning rate benefit exhibited in the rotation group is a consequence of a deeper learning of rotation structure rather than the formation of an aiming heuristic which generalizes to other perturbation structures.

The finding that structural learning is highly dependent on the statistics of the environment raises the question of whether transfer between perturbation structures is possible. Previous work has shown that learning a single gain perturbation at distal targets eliminates direction-specificity in rotation generalization, leading to rotation generalization across the entire workspace (Yin et al., 2016). It is unclear if this finding is consistent with a form of structural learning or a more general change in sensitivity to any form of visuomotor error. The source of transfer between different perturbations remains an open question. For example, it could be that a series of shear perturbations, which would require a remapping of both the extent and direction of the movement vector to restore task performance, would improve both gain and rotation learning. Furthermore, this effect could be unidirectional such that gain or rotation structure training does not improve shear learning. Alternatively, structural learning may not necessarily be confined by the mathematical similarities between perturbations. Instead, the similarity between adapted responses to given perturbation types may dictate the degree to which participants generalize between structures. For example, the adaptive responses to a shear and a rotation are much more similar than the responses to a gain and a shear; simply angling the limb to offset the perturbation would aid performance in both of the former perturbations. Further work is necessary to test the specificity of generalization between different structures.

## Conclusions

Overall, our results provide further support for the general consensus that explicit re-aiming is an essential component of sensorimotor learning in a visuomotor rotation task and may be responsible for a variety of motor learning behaviors thought to be largely implicit (Heuer and Hegele, 2008; Hegele and Heuer, 2010; Taylor et al., 2014; Bond and Taylor, 2015; McDougle et al., 2015; Morehead et al., 2015; Brudner et al., 2016; Day et al., 2016; Poh et al., 2016). There are two primary implications of our results. First, because rotation structure learning is explicitly accessible, it may share a common learning mechanism with rule-based learning in other domains which rely on abstraction, such as category learning and concept learning. Correspondingly, it may recruit neural systems associated with explicit rule formation in support of adaptive behavior, such as the prefrontal cortex and striatum.

**Table 3.** A summary of statistical analyses

*Line*	*Dependent variable*	*Test*	*Statistic*	*Confidence*
a	Average endpoint hand angles during last epoch of the baseline phase for all groups in experiment 1	Two-way ANOVA	Structure_*F*_(1,36)_ = 0.60, report_*F*_(1,36)_ = 4.12, interaction_*F*_(1,36)_ = 0.20	Structure_partial η^2^ = 0.01, *p* = 0.4441; report_partial η^2^ = 0.1, *p* = 0.0498; interaction_partial η^2^ = 0.01, *p* = 0.6579
b	Average endpoint hand angles for nostructure-noreport and nostructure-report groups during last epoch of the baseline phase and average endpoint hand angles for the exposure phase	Paired *t* test	Nostructure-noreport_*t*_(9)_ = -0.77	Nostructure-noreport _CI: −0.8441/0.4141, *p* = 0.4593; nostructure-report_CI: 0.2853/2.9324, *p* = 0.0225
			Nostructure-report_*t*_(9)_ = 2.75	
c	Correlation coefficients for exposure phase endpoint hand angles and rotation solutions for structure-report and structure-noreport groups	Two-sample *t* test	*t*_(18)_ = 2.94	CI: 0.0699/
				0.4207, *p* = 0.0088
d	Endpoint hand angle-solution regression slopes for structure-report and structure-noreport groups	One-sample *t* test	Structure-report_*t*_(9)_ = 14.90, structure-noreport_*t*_(9)_ = 7.40	Structure-report _CI: 0.6139/0.8336, *p* = 1.1967e-07; structure-noreport_CI: 0.3582/0.6735, *p* = 4.0938e-05
e	Endpoint hand angle-solution regression slopes for structure-report and structure-noreport groups	Two-sample *t* test	*t*_(18)_ = 2.45	CI: 0.0294/0.3864, *p* = 0.0249
f	Aiming angle-solution regression slope for structure-report group	One-sample *t* test	*t*_(9)_ = 13.31	CI: 0.6063/0.8546, *p* = 3.1703e-07
g	Implicit angle-solution regression slope for structure-report group	One-sample *t* test	*t*_(9)_ = −0.12	CI: −0.0463/0.0418, *p* = 0.9106
h	Average endpoint hand angles for all groups in experiment 1 during last epoch of the feedback-washout phase	Two-way ANOVA	Structure_*F*_(1,36)_ = 5.31, report_*F*_(1,36)_ = 4.17, interaction_*F*_(1,36)_ = 6.54	Structure_partial η^2^ = 0.13, *p* = 0.0271; report_partial η^2^ = 0.10, *p* = 0.0484; interaction_partial η^2^ = 0.15, *p* = 0.0149
i	Average aiming angles for structure-report and nostructure-report groups during last epoch of the feedback-washout phase	Two-sample *t* test	*t*_(18)_ = −1.41	CI: −0.2087/1.0525, *p* = 0.1769
j	Average endpoint hand angles during first epoch of the test phase for all groups in experiment 1	Two-way ANOVA	Structure_*F*_(1,36)_ = 36.28, report_*F*_(1,36)_ = 0.21, interaction_*F*_(1,36)_ = 2.38	Structure_partial η^2^ = 0.50, *p* = 6.48e-07; report_partial η^2^ = 0.01, *p* = 0.648; interaction_partial η^2^ = 0.06, interaction_ *p* = 0.132
k	Average aiming angles and implicit angles during first epoch of the test phase for structure-report and nostructure-report groups	One-way MANOVA	*F*_(1,18)_ = 24.60	Pillai’s trace = 0.74, *p* = 9.582e-06
l	Baseline-subtracted, average endpoint hand angles during first epoch of the no-feedback washout phase for all groups	Two-way ANOVA	Structure_*F*_(1,36)_ = 3.55, report_*F*_(1,36)_ = 0.57, interaction_*F*_(1,36)_ = 0.55	Structure_partial η^2^ = 0.09, *p* = 0.0677; report_partial η^2^ = 0.02, *p* = 0.4541; interaction_partial η^2^ = 0.02, *p* = 0.4620
m	Average endpoint hand angles in experiment 2 during last epoch of the baseline phase for all groups	One-way ANOVA	*F*_(2,39)_ = 0.76	Partial η^2^ = 0.04, *p* = 0.4764
n	Average endpoint hand radii in experiment 2 during last epoch of the baseline phase for all groups	One-way ANOVA	*F*_(2,39)_ = 4.44	Partial η^2^ = 0.19, *p* = 0.0182
o	Average control group endpoint hand angles during last epoch of the baseline phase and exposure phase	Paired *t* test	*t*_(13)_ = 2.64	CI: 0.2869/2.8518, *p* = 0.0203
p	Correlation coefficients for exposure phase reach performance and perturbation solutions for gain and rotation groups	Two-sample *t* test	*t*_(26)_ = −2.10	CI: 0.0034/0.3221, *p* = 0.0456
q	Exposure phase reach-solution regression slopes for gain and rotation groups	One-sample *t* test	Gain_*t*_(13)_ = 6.88,	Gain_CI: 0.2296/0.4396, *p* = 1.1109e-05; rotation_CI: 0.3739/0.6463, *p* = 1.9788e-06
			Rotation_*t*_(13)_ = 8.09	
r	Exposure phase reach-solution regression slopes for gain and rotation groups	Two-sample *t* test	*t*_(26)_ = −2.20	CI: −0.3391/−0.0119, *p* = 0.0365
s	Average endpoint hand angles for all groups in experiment 2 during last epoch of the feedback-washout phase	One-way ANOVA	*F*_(2,39)_ = 1.3	Partial η^2^ = 0.06, *p* = 0.2850
t	Average aiming angles for all groups in experiment 2 during last epoch of the feedback-washout phase	One-way ANOVA	*F*_(2,39)_ = 5.63	Partial η^2^ = 0.22, *p* = 0.0071
u	Average endpoint hand radii for all groups in experiment 2 during last epoch of the feedback-washout phase	One-way ANOVA	*F*_(2,39)_ = 0.68	Partial η^2^ = 0.03, *p* = 0.5109
v	Average aiming radii for all groups in experiment 2 during last epoch of the feedback-washout phase	One-way ANOVA	*F*_(2,39)_ = 0.79	Partial η^2^ = 0.04, *p* = 0.4608
w	Average endpoint hand angles for all groups during first epoch of the test phase	One-way ANOVA	*F*_(2,39)_ = 15.36	Partial η^2^ = 0.44, *p* = 1.2049e-05
x	Reaching radii for all groups during first epoch of the test phase	One-way ANOVA	*F*_(2,39)_ = 0.11	Partial η^2^ = 0.01, *p* = 0.8956
y	Aiming radii for all groups during first epoch of the test phase	One-way ANOVA	*F*_(2,39)_ = 0.19	Partial η^2^ = 0.01, *p* = 0.8317
z	Average aiming angles for all groups during first epoch of the test phase	One-way ANOVA	*F*_(2,39)_ = 10.90	Partial η^2^ = 0.36, *p* = 1.7326e-04
aa	Average implicit angles for all groups during first epoch of the test phase	One-way ANOVA	*F*_(2,39)_ = 0.99	Partial η^2^ = 0.05, *p* = 0.3816
bb	Average aiming angles and average endpoint hand angles during first epoch of test phase	Paired *t* tests	Rotation_*t*_(13)_ = 0.35,	Rotation_CI:−5.0710/3.6744, *p* = 0.7356; gain_CI: −6.6180/15.0414, *p* = 0.4160; control_CI: −4.9148/1.2700, *p* = 0.2253
			gain_*t*_(13)_ = −0.84,	
			control_*t*_(13)_ = 1.27	
cc	Average aiming angles and average endpoint hand angles during first epoch of test phase	Pearson correlations	Rotation_*r* = 0.78,	Rotation_CI: 0.4269/0.9272, rotation_*p* = 0.0010; gain_CI: 0.5874/0.9523, *p* = 0.0001; control_CI: 0.7427/0.9726, *p* = 5.0555e-06
			gain_*r* = 0.85,	
			control_*r* = 0.91	

The first column specifies the superscript letter used to identify the statistical test within the manuscript, the second column lists the dependent variable on which the test is conducted, the third column lists the type of test used, the fourth column shows the test statistic, and the final column provides a measure of confidence appropriate for the type of test conducted.

## Synthesis

Reviewing Editor: Lila Davachi, New York University Decisions are customarily a result of the Reviewing Editor and the peer reviewers coming together and discussing their recommendations until a consensus is reached. When revisions are invited, a fact-based synthesis statement explaining their decision and outlining what is needed to prepare a revision will be listed below. The following reviewer(s) agreed to reveal their identity: Nicolas Schweighofer, Denise Henriques

Reviewer 1

This well-written paper 1) replicates structural learning phenomenon for visuomotor rotations using as controls either no pre-exposure or non-congruent (gain) adaptation, and 2) uses solid 2x2 designs to show that most of the gains in re-adaptation after random exposure are due to explicit aiming components.

Major:

The authors cite Morehead et al. 2015 to write that “explicit re-aiming is entirely responsible for savings”. However, the Morehead et al. study also show that savings occur for large but not small perturbations. Here, the mean of the absolute value of the perturbations in the exposure phase is near 45 degree (a bit less since 60 degree rotations were not presented). So the question is whether the phenomenon reported also occurs for smaller perturbations in the exposure phase? My guess is that it is not, and that the results of the present study are thus an extension of previous results showing that savings following exposure to large perturbations. At the very least, the difference between large and small perturbations should be discussed. At best, the study should include a new group with small random perturbations in the exposure phase.

Minor:

The main results depend on average of first 8 trials in re-learning (60 degree perturbation phase). Thus, the results depend on the number of trials used (8). Please consider additional parameter analyses without free parameters, such as exponential fits (see for example Huang et al. 2011), or at least show that results do not overly depend on magic number 8.

The two-stage regressions may lack power. If I understand well, a regression between the actual adaptation and ideal adaptation was fitted for each participant; then slopes were compared to 0 and/or between groups. Authors should consider linear mixed effect models to fit a single model to all subjects and groups to test significance and difference in slopes.

It would be helpful to provide a roadmap of what is tested in experiments 1 and 2. The reader only becomes aware of the differences as s/he is reading along text in experiment 2.

It is unclear until later in the results whether the schedule of pseudo-random perturbations in the exposure phase is the same or different for all subjects.

In each figure, it would be useful to refer to experiment 1 or 2.

I am not sure that tables 1 and 2 add much to the study. Consider removing. In table 3, I would only show the most important results, or remove since these results are in text anyway.

Savings can also be due to true meta-learning, increased learning rates. See and discuss work by Herzfeld et al. 2014.

Reviewer 2:

This study explores the nature of the structural learning on visuomotor adaptation. Mainly it assesses the contribution of explicit and implicit processes on the structural learning and its resulting benefits on savings, or learning rate of a novel but related perturbation. It does so using the Ivry & Taylor aiming tasks for each trial, i.e., the report groups, along with groups that do not aim to ensure that the reporting doesn't contribute to learning/savings.

The first experiment shows that structural learning via experience with variable cursor rotations leads to greater savings in a novel rotation compared to after training only with a veridical cursor. This was only slightly larger in the reporting group. But all of the changes in reach direction, both during training with the random cursor and during learning the novel cursor rotation afterward reflected mainly changes in explicit processes (re-aiming).

This was also the case for the second experiment which showed greater savings during the novel cursor rotation following exposure with rotated cursor motion compared to scaled cursor motion (as well as veridical cursor motion). Not surprisingly, the greater savings was only seen when the various cursor perturbation was also rotated.

I think the experiments are very nicely designed and the analysis careful and thorough, and the whole paper is well written. My only “reservation” is that the question and findings are not entirely novel or surprising. While directly measuring the contribution of explicit strategy hasn't been done, I don't think the results showing that changes in reach direction during, and faster learning following, exposure to variable related perturbations is surprising. I would have been more shocked to find any implicit adaptation, especially in the exposure part. I also believe that Braun et al referred to their original findings regarding the benefits of structural learning on subsequent learning rate as “strategic adaptation”. My own understanding of structural learning is figuring out the “general” rule to apply or initially explore. So while the results of this paper clearly demonstrate that structural learning and its resulting effect on learning rate are explicit, I don't find any of this such a surprise.

Even the additional question addressed in the second experiment doesn't seem very novel. As previous studies on this topic have shown, structural learning is based on the fact that the parameters and thus the “rule” that needs to be applied to during subsequent testing should similar. I don't think experiencing a gain would qualified of structural learning for adaptation of a rotation; the parameters are not the same. The heuristic hypothesis is a bit of a straw-man.

Plus, a cursor-gain is likely something that all of us computer users have already “structurally learned” through using a mouse (for different devices, or even different speeds with the same mouse). Our life-long structural experience with cursor gains probably explains the original finding by Krakauer et al (2000) that we learn faster and generalize broader cursor gains than cursor rotations.
